# Change in children’s school behavior after mass administration of praziquantel for *Schistosoma mansoni* infection in endemic areas of western Kenya: A pilot study using the Behavioral Assessment System for Children (BASC-2)

**DOI:** 10.1371/journal.pone.0181975

**Published:** 2017-07-26

**Authors:** Rosemary Musuva, Ye Shen, Xianjue Wei, Sue Binder, Julianne A. Ivy, W. Evan Secor, Susan P. Montgomery, Charles H. King, Pauline N. M. Mwinzi

**Affiliations:** 1 Centre for Global Health Research, Kenya Medical Research Institute, Kisumu, Kenya; 2 Department of Epidemiology & Biostatistics, University of Georgia, Athens, Georgia, United States of America; 3 Schistosomiasis Consortium for Operational Research and Evaluation, University of Georgia, Athens, Georgia, United States of America; 4 Center for Global Health and Diseases, Case Western Reserve University, Cleveland, Ohio, United States of America; 5 Parasitic Diseases Branch, Division of Parasitic Diseases and Malaria, Centers for Disease Control and Prevention, Atlanta, Georgia, United States of America; Johns Hopkins University, UNITED STATES

## Abstract

**Background:**

Schistosomiasis is a parasite-related chronic inflammatory condition that can cause anemia, decreased growth, liver abnormalities, and deficits in cognitive functioning among children.

**Methodology/Principal findings:**

This study used the Behavior Assessment System for Children (BASC-2) to collect data on thirty-six 9–12 year old school-attending children’s behavioral profiles in an *Schistosoma mansoni*-endemic area of western Kenya, before and after treatment with praziquantel for *S*. *mansoni* infection. BASC-2 T scores were significantly reduced post-treatment (p < 0.05) for each of the ‘negative’ behavior categories including externalizing problems (hyperactivity, aggression, and conduct problems that are disruptive in nature), internalizing problems (anxiety, depression, somatization, atypicality, and withdrawal), school problems (academic difficulties, included attention problems and learning problems), and the composite behavioral symptoms index (BSI), signifying improved behavior. While the observed improvement in the ‘positive’ behavior category of adaptive skills (adaptability, functional communication, social skills, leadership, and study skills) was not statistically significant, there were significant improvements in two adaptive skills subcategories: social skills and study skills.

**Conclusion/Significance:**

Results of this study suggest that children have better school-related behaviors without heavy *S*. *mansoni* infection, and that infected children’s behaviors, especially disruptive problem behaviors, improve significantly after praziquantel treatment.

## Introduction

Schistosomiasis is an infectious disease caused by trematode parasites (blood flukes) of the genus *Schistosoma*. This disease is an important global cause of chronic morbidity, including anemia, stunted growth, and malnutrition [1]. The World Health Organization (WHO) states that at least 258 million people worldwide needed treatment for schistosomiasis in 2014; however, only 61.6 million actually received it [2]. Schistosomiasis is prevalent in many tropical and subtropical regions [3], and it is particularly common in western Kenya, where prevalence of egg-positive *Schistosoma mansoni* infection approaches 70% in many local schools [4]. In such settings, childhood exposure to *Schistosoma* infection is nearly universal [5], and can affect growth and development from an early age [6].

Preventing exposure to schistosomiasis is difficult in many resource-limited areas where the disease is now endemic. Because of this, the currently recommended approach to decreasing schistosomiasis-related morbidity is to offer routine mass drug administration (MDA) with praziquantel to all school-age children and at-risk adults, regardless of their symptoms or egg test status [7]. Low intensity infections often get missed by standard coproscopic testing [8], so that mass treatment is currently the best means to reach all infected residents. When implemented correctly, this strategy has been shown to markedly reduce prevalence of organ-specific *Schistosoma-*related disease [9–12], but its impact on concurrent systemic morbidities has not been well studied [13]. Specifically, it has been recognized that the presence of schistosomiasis can cause a number of non-specific but potentially very significant functional pathologies, including impairment of children’s memory, learning, and school performance [14], with resultant losses of local human capital due to deficiencies in education [1].

The pathology of schistosomiasis is associated with prolonged host inflammatory responses to parasite eggs, and this chronic inflammation provokes cellular dysfunction throughout the body. Previous studies have associated schistosomiasis with anemia of chronic inflammation, undernutrition, growth stunting, loss of fitness, and impaired cognitive function [14–18]. Because other chronic inflammatory processes have been causally linked to psychological disease manifestations [19, 20], for the present study, we chose to examine the impact of *S*. *mansoni* infection on at-risk schoolchildren’s behavior. To do this, we employed an established, standardized survey tool, the Behavior Assessment System for Children, 2^nd^ edition (BASC-2) [21, 22].

The BASC-2 is an in-depth multidimensional survey instrument created to evaluate behavioral and emotional problems in school-aged children [21, 22]. The present study employed the BASC-2 evaluation based on Teacher Rating Scales (TRS), in which teachers relate, in a structured format, how frequently students exhibit certain normal and abnormal behaviors [21]. Their responses are then converted into standardized scores, which can then be clinically classified and compared [21]. In our study, the BASC-2 was used to evaluate the behavior of *S*. *mansoni* egg-positive and egg-negative schoolchildren in *S*. *mansoni-*endemic areas of western Kenya, both before and after praziquantel drug therapy. In this setting, praziquantel treatment would be expected to uniquely affect *Schistosoma* infection alone. By formally assessing the differences in behavior pre-and post-treatment, we aimed to test the utility of the BASC-2 instrument in elucidating the impact that chronic *S*. *mansoni* infection could have on children and their in-school functioning.

## Materials and methods

### Data collection

#### Ethics statement

This study was approved by the Scientific Steering Committee and Ethical Research Committee (ERC) of the Kenya Medical Research Institute (KEMRI). The protocol was also reviewed at the U.S. Centers for Disease Control and Prevention (CDC), which elected to defer study ethical oversight to the KEMRI ERC. Thereafter, permission was obtained from the Provincial Administration and the subjects’ local health provider, the Ministry of Public Health and Sanitation (MoPHS). The purpose of the study and its objectives were explained to participating teachers and written parental consent and child assent were obtained at enrollment. The mass drug administration praziquantel treatments described in this paper were performed as part of a concurrent registered clinical trial, ISRCTN16755535 (doi 10.1186/ISRCTN16755535), “Comparison of school and community-based MDA delivery strategies for control of *Schistosoma mansoni* infections in western Kenya in areas with >25% prevalence”, which was jointly performed by KEMRI and CDC investigators [23].

#### Student parasitological testing and selection for evaluation

*S*. *mansoni* prevalence surveys were conducted in January 2014, then a total of 36 children from five grade levels in six different schools were selected for this pilot study. These schools were in Rarieda and Bondo districts in villages near the shores of Lake Victoria where the prevalence of *S*. *mansoni* is very high. In 2014, there were at least 15 children in each of these schools’ classes with ≥ 400 eggs/gram feces, as detected using the Kato-Katz (KK) thick smear technique [24]. For the present study, six children from each school were selected from two specific infection strata: three children were randomly selected from children with no detectable eggs in their stools (egg-negative infection status based on examination of three consecutive daily stools, with two slides prepared per stool [24]). Another three children were randomly selected from those with an average of at least 400 eggs per gram feces (egg-positive, heavy infection status) in their three daily stool specimens.

#### Participating teacher/assessors

Six teachers of classes having 8–11 year old students were asked to participate. During the BASC-2 assessment process, these teacher/assessors and field teams were blinded as to which of the participating children were egg-positive and which had no eggs in their stool.

A one day training was held with the teachers to introduce the behavioral study and its objectives, and to provide a thorough understanding of the BASC-2 teacher rating scale that was to be administered. After training by the research team, teachers used the BASC-2 teacher rating questionnaire forms to evaluate the 36 study children at a time point three weeks prior to initiation of MDA in order to collect the before-treatment data. MDA occurred in February 2014, and three weeks following the MDA, the same teachers re-evaluated the same children using the BASC-2 to collect the after-treatment data. MDA treatment consisted of a standard FDA-approved oral dose of the anti-helminthic drug, praziquantel, given at 40 mg/kg body weight [25]. Changes in BASC-2 scoring between the two time points were then evaluated.

#### The BASC-2 instrument

The Behavior Assessment System for Children, 2^nd^ edition (BASC-2, John Wiley & Sons, Inc. available at http://onlinelibrary.wiley.com) is a widely used, broad-based behavioral rating survey used by educational practitioners [22]. Either sex-based norms or combined-sex norms can be used in scoring of BASC-2 surveys, and this study used the latter [21]. There are also three types of scales that can be used in performing the BASC-2: Teacher Rating Scales (TRS), Parent Rating Scales (PRS), or the Self-Report of Personality (SRP) [21]. To obtain the greatest observer consistency, we elected to use the Teacher Rating Scales to evaluate the participating children’s school behaviors in this study. The survey was conducted in English, in which the teachers were fully proficient.

Briefly, the TRS is a measure of both adaptive and problematic behaviors in the school setting. It has forms for three age levels: preschool (2 through 5), child (6 through 11), and adolescent (12 through 21) [21]. For our study, the ‘child’ scale, TRS-C, was used, as enrolled students were ages 8 through 11. We used a total of 129 questions from the TRS, each asking about frequency of specific behaviors. Teachers responded to these questions with ‘Never’, ‘Sometimes’, ‘Often’, or ‘Almost Always’, which, for analysis, were converted to corresponding scores of 1, 2, 3, and 4.

The analysis of BASC-TRS results yielded 5 composite scales, 10 clinical scales, and 5 adaptive scales. The broader composite scales were: externalizing problems, internalizing problems, school problems, adaptive skills, and the aggregate Behavioral Symptoms Index (BSI) [21]. ‘Externalizing problems’ contained data inputs about hyperactivity, aggression, and conduct problems that are disruptive in nature. ‘Internalizing problems’ covered behaviors that did not involve acting-out, including anxiety, depression, somatization, atypicality, and withdrawal. The ‘school problems’ score, reflecting academic difficulties, included attention problems and learning problems. ‘Adaptive skills’ had five subscales: adaptability, functional communication, social skills, leadership, and study skills. The BSI was an overall category that summed problem behaviors, including hyperactivity, aggression, depression, atypicality, withdrawal, and attention problems [21].

To measure the validity of teachers’ responses, there were three monitoring indices: the F-index, the Consistency index, and the Response Pattern index. The F-index score was a tally of the number of times the respondent provided a very negative behavior rating [21]. The Response Pattern index was designed to identify forms that might be invalid due to respondent inattention to the item content [21]. The Consistency index identified cases in which the respondent offered inconsistent answers to items that usually are answered similarly [21]. If the responses were consistent, the value of Consistency index was considered “acceptable”; otherwise, the Consistency index was listed as “caution”. The Response Pattern index was acceptable for all the observations in our study, so we have only reported on analysis of the F-index and the Consistency index.

BASC-2 ASSIST Software (Pearson Clinical, Bloomington, MN, USA; available at http://www.pearsonclinical.com) [21, 22] was used to preprocess the raw data collected from the BASC-2 TRS-C questionnaires and to generate summary T scores. These were then entered into a working dataset (see supporting information S2 File) for further statistical analysis.

### Statistical analysis

Of the 36 children enrolled, half (n = 18) were *S*. *mansoni* egg-positive and the other half egg-negative based on standard KK stool testing. BASC-2 TRS-C information for each child was collected before and after MDA treatment, with the expectation of 72 observations. However, there was one egg-positive child for whom only before-treatment data were recorded. Hence, 71 observations were included in the complete-case analysis and a multiple imputation approach (explained in detail below) was used to serve as a sensitivity check in the follow-up sensitivity analysis of the potential impact of missing data.

The numerical values (1, 2, 3, and 4) for each question were entered into the BASC-2 software manually, and the software generated T scores for each survey. For the following categories, a higher BASC-2 T score indicated more frequent problematic behaviors: externalizing problems, internalizing problems, school problems, and the BSI (an overall score for behavior problems). For adaptive skills, a higher T score indicated more frequent positive or favorable behaviors. The resulting T score data were analyzed using SAS version 9.4 (SAS Institute, Inc., Cary, NC). To assess the relative impact of heavy *S*. *mansoni* infection, the mean scores for externalizing problems, internalizing problems, school problems, adaptive skills, and BSI were also calculated according to *S*. *mansoni* egg status (i.e., stool egg-negative or egg-positive). All tests and confidence intervals used the 5% level of significance.

Composite BASC-2 T scores were further classified into three ranges: ‘average’, ‘at risk’, and ‘clinically significant’. For externalizing problems, internalizing problems, school problems, and the Behavioral Symptoms Index, T scores from 41 to 59 were considered average, 60–69 were considered at-risk, and 70 or above were considered clinically significant based on BASC-2 guidelines [21]. Descriptive analysis was also conducted for the subscales of each composite scale. For adaptive skills, scores of 41–59 were considered average, 31–40 were considered at-risk, and 30 or below were considered clinically significant [21].

Normality checking for the dataset was done using quantile-quantile (QQ) plots by time and egg-status, respectively, to ensure that methods relying on normal distributional assumptions were valid [26]. To test the hypothesis on whether there is a significant difference in the composite T scores between before-treatment and after-treatment, a paired two-sample Student’s t-test was conducted. Multi-level linear mixed effects models were built for each composite scale to further adjust for potential confounding and explore the change in composite T scores following treatment among children with egg-positive status, as compared to those with egg-negative status. Since the F-Index and Consistency Index for some children changed after the treatment, McNemar’s tests were conducted to test for the presence of significant differences in these indices following MDA. The original BASC-2 output reported three levels of the F-Index and the Consistency Index: ‘acceptable’, ‘caution’, and ‘extreme caution’. In our study, we combined the caution and extreme caution categories together as flags for special attention, and created 2 x 2 concordance tables for performance of the McNemar’s Test.

#### Sensitivity analysis by multiple imputation

Because there were missing data in our study, we attempted to statistically address the missingness as a sensitivity check. Multiple imputation can provide unbiased statistical results given a correctly specified imputation model [27]. The approach we adopted, so-called multiple imputation (MI), assumed that missing values were missing at random (MAR) in which the probability that a datum was missing might have depended on observed characteristics but not on unobserved characteristics of the subject [28]. In our analysis, considering that we had both continuous and categorical variables to be imputed and that an arbitrary missing data pattern allows more flexibility over a monotone missing pattern, we applied the fully conditional specification (FCS) approach, which was based on a flexible selection of univariate imputation distributions, without formally specifying the joint multivariate density [29].

## Results

### Study population characteristics

The demographic characteristics of the study participants are shown in Table 1. By design, the egg-positive and egg-negative study subgroups were quite similar for sex ratio, age, class assignment and schools attended (Table 1).

**Table 1:** Demographic characteristics of study groups.

Variable	Egg-negative group (N = 18)	Egg-positive group (N = 18)
**M:F ratio**	1:1	1.25:1
**Mean age ± SD (range)**	9.7 ± 0.75 (8–11 yr)	10.0 ± 0.78 (9–11 yr)
**Children per grade level**		
Grade 2	3	3
Grade 3	3	3
Grade 4	8	8
Grade 5	3	3
Grade 6	1	1
**Children per school**		
Lenrose	3	3
Luore Primary	3	3
Magawa Primary	3	3
Orengo	3	3
Ranyala	3	3
Uyawi	3	3

### BASC-2 assessments before and after MDA

**Fig 1:**
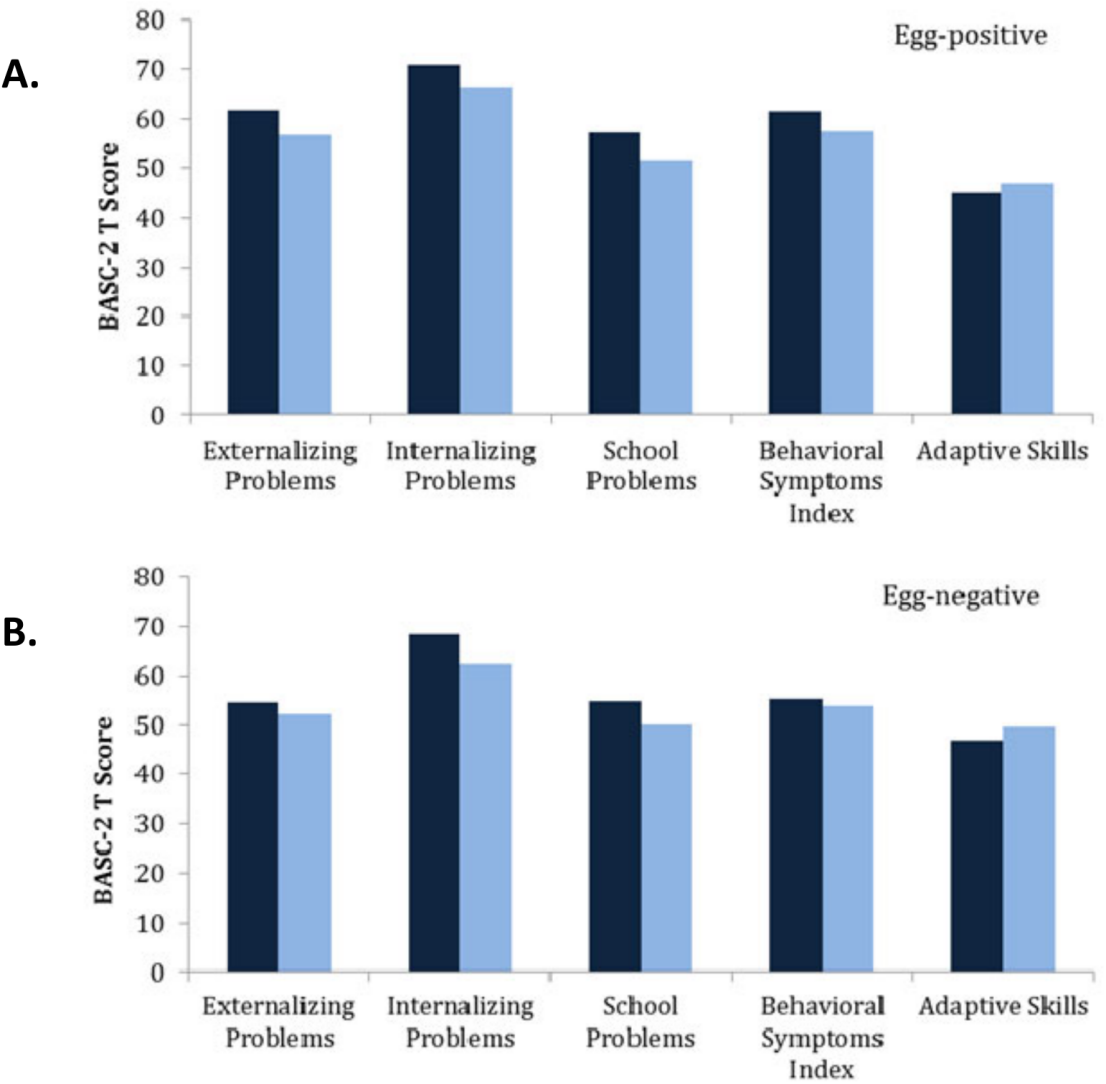
**Mean BASC-2 T scores for *S*. *mansoni* egg-positive (Panel A) and egg-negative (Panel B) students before and after treatment with praziquantel.** The results of the BASC-2 survey were transformed into standardized T scores for each of 5 composite behavior categories. The mean T scores for each group of students were then determined; the numerical values and standard deviations are listed in S1 Table. A lower score indicates fewer problematic behaviors for externalizing problems, internalizing problems, school problems, and the Behavioral Symptoms Index. A higher score indicates more favorable behaviors for Adaptive Skills. dark blue = before treatment; light blue = after treatment.

In terms of the students’ behavior assessments, Fig 1 and supplemental S1 Table show the mean students’ T scores before and after praziquantel MDA treatment, according to their pre-treatment infection category.

Among all students taken together (N = 35), BASC-2 T scores were significantly reduced post-treatment (p < 0.05, Table 2) for each of the ‘negative’ behavior categories including externalizing problems (hyperactivity, aggression, and conduct problems that are disruptive in nature), internalizing problems (anxiety, depression, somatization, atypicality, and withdrawal), school problems (academic difficulties, included attention problems and learning problems), and the BSI, signifying improved behavior. While the observed improvement in the ‘positive’ behavior category of adaptive skills (adaptability, functional communication, social skills, leadership, and study skills) was not statistically significant, there were significant improvements in two adaptive skills subcategories: social skills and study skills (Table 3).

**Table 2:** Student’s paired t-test of the average difference in BASC-2 T scores from before MDA treatment to after treatment for all participating students (N = 35).

Variable	Mean change (before—after)	Std. error of the mean	t	d.f.	p-value
**Externalizing Problems**	4	1.34	2.97	34	0.0054*
**Internalizing Problems**	5.37	2.07	2.59	34	0.0139*
**School Problems**	5.26	1.35	3.91	34	0.0004*
**Behavioral Symptoms Index**	2.89	1.37	2.11	34	0.0425*
**Adaptive Skill**	-2.43	1.34	-1.81	34	0.0784

* indicates p < 0.05

**Table 3:** Student’s paired t-test of the average difference in adaptive skills subcategory T scores for all participants (n = 35) before treatment vs. after treatment.

Variable	Mean change (before—after)	Std. error of the mean	t	d.f.	p-value
**Adaptability**	0.09	1.26	-0.07	34	0.9462
**Social Skills**	-4.29	1.76	2.44	34	0.0200*
**Leadership**	-2.69	1.56	1.73	34	0.0940
**Study Skills**	-2.74	1.31	2.09	34	0.0444*
**Functional Communication**	-0.77	1.79	0.43	34	0.6689

* indicates p < 0.05

### Analysis by egg count status

When broken down by infection status, mean T scores for negative behavior categories were consistently higher (*i*.*e*., worse) in the *S*. *mansoni* egg-positive heavy infection group compared to the egg-negative group (S1 Table). However, these differences between groups were only significant for the externalizing problems category, when scored prior to MDA treatment (p = 0.042). For adaptive skills, mean T scores were lower (worse) in the egg-positive heavy infection group compared to the egg-negative group, but not at a statistically significant level.

In multivariable analysis, in which the multiply-adjusted impact of time, infection, and age on the BASC-2 T scores was evaluated (Table 4), the time factor (i.e., whether before or after treatment) proved to have a significant (p < 0.05) impact on four of the five behavior categories–adaptive skills was the exception. *S*. *mansoni* stool test status (egg-positive or egg-negative) was also associated with an independent significant effect on externalizing problems and on the Behavioral Symptoms Index. There was no significant age effect observed, and there was no significant interaction between egg status and the timing of the examination.

**Table 4:** Multiply-adjusted individual effects of time, egg-positivity status, age, or the interaction of time and infection status on BASC-2 T scores.

Scales	Effect	Num DF	Den DF	F Value	P-value
**Externalizing Problems**	**Time**	1	33	8.32	0.0069*
	**Status**	1	33	7.16	0.0115*
	**Age**	1	33	2.06	0.1604
	Time*Status	1	33	1.13	0.2957
**Internalizing Problems**	**Time**	1	33	6.9	0.0129*
	**Status**	1	33	0.44	0.5124
	**Age**	1	33	3.58	0.0674
	Time*Status	1	33	0.2	0.6542
**School Problems**	**Time**	1	33	14.74	0.0005*
	**Status**	1	33	0.46	0.5039
	**Age**	1	33	1.92	0.1746
	Time*Status	1	33	0.06	0.8156
**Behavioral Symptoms Index**	**Time**	1	33	4.15	0.0497*
	**Status**	1	33	4.36	0.0447*
	**Age**	1	33	1.84	0.1845
	Time*Status	1	33	0.91	0.3475
**Adaptive Skills**	**Time**	1	33	3.11	0.0872
	**Status**	1	33	1.13	0.2949
	**Age**	1	33	0.94	0.3392
	Time*Status	1	33	0.13	0.7242

* indicates p < 0.05

### Individual classification of BASC-2 scores

**Fig 2:**
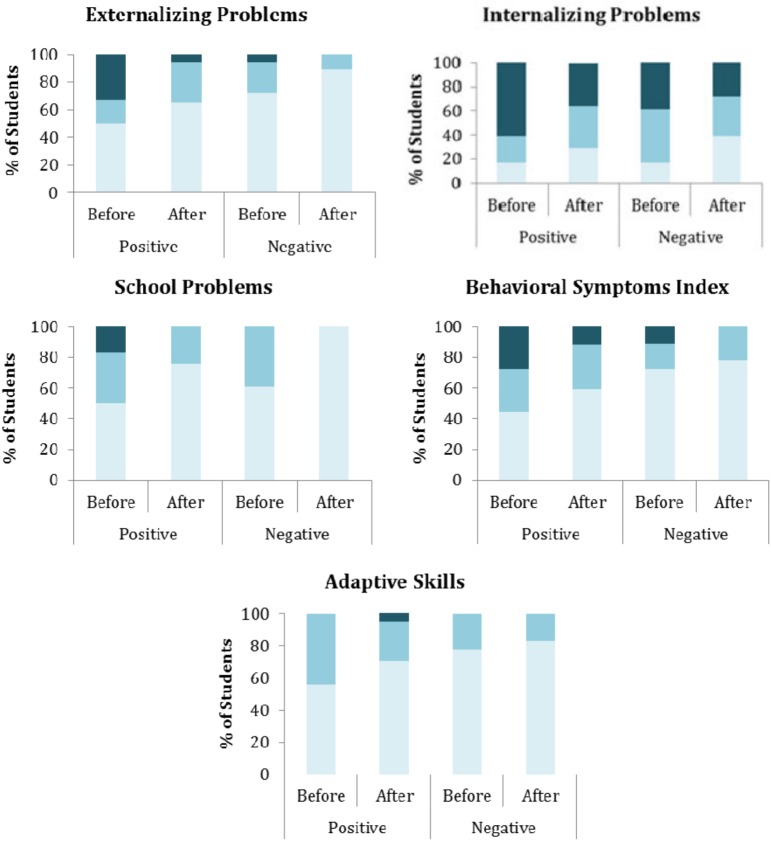
Percent of students with average, at-risk, or clinically significant BASC-2 T scores. Percentages were calculated for *S*. *mansoni* egg-positive and egg-negative students both before and after treatment. Color intensity represents the category: dark blue = clinically significant; medium blue = at-risk; light blue = average.

Following BASC-2 classification guidance, the behavioral assessment scores were translated into clinically relevant descriptors [21]. Fig 2 shows the percentage of students placed into each classification for each behavior category. In all cases, the number of students in the “average” range increased after treatment. Also, for all categories except adaptive skills, the number of students in the “clinically significant” range decreased after treatment.

### Assessment of the relative effect size of BASC-2 changes observed after MDA

Cohen’s effect size provided another method of analyzing the impact of MDA on study subjects’ BASC-2 behavior scores (S2 Table–S5 Table). Effect sizes were determined based on the standardized differences between two groups (i.e. before vs. after treatment), where a calculated effect size of d = 0.2 to 0.49 was considered small; d = 0.5 to 0.79 was considered a medium size effect; and d ≥ 0.8 was considered a large effect. By these criteria, when evaluating the effect of treatment on the combined student groups, there was a medium sized beneficial effect on externalizing problems and school problems scores. In the egg-positive heavy infection group by itself (S4 Table), there was a large effect of treatment on school problems and a medium effect on externalizing problems and the summary BSI. In the egg-negative group (S5 Table), there was a medium-sized effect of treatment on internalizing problems and school problems.

### Validity indices

There are two important indices used to validate BASC-2 survey results: The F-Index aims to test whether the distribution is highly skewed, suggesting a floor or a ceiling effect in the data, and the Consistency Index evaluates whether respondents’ answers were consistent for questions within the same domain. Table 5 indicates that there were a number of surveys with a “caution” level F-index before and/or after the treatment. However, the before vs. after McNemar’s test statistic for the F-Index was 0.0769, with a corresponding p-value close to 1, which suggested that there was no significant change in BASC-2 questionnaire performance from before treatment to after treatment.

**Table 5:** F-index category distributions when the BASC-2 scale was administered before and after praziquantel mass treatment^a^.

		After Treatment	
		Acceptable	Caution
**Before Treatment**	Acceptable	19	6
	Caution	7	3

^a^ McNemar’s test statistic = 0.0769; p = 1

A parallel analysis was performed for the Consistency Index. Table 6 shows that more surveys switched from “caution” to “acceptable” status after treatment than the other direction. McNemar’s Test statistic for the Consistency Index is 8.0667 with a corresponding p-value of 0.0074. This suggested that the survey responses showed more internal consistency after treatment than before treatment.

**Table 6:** Consistency index category distributions when the BASC-2 scale was administered before and after praziquantel mass treatment^a^.

		After Treatment	
		Acceptable	Caution
**Before Treatment**	Acceptable	8	2
	Caution	13	12

^a^ McNemar’s test statistic = 8.0667; p = 0.0074

### Sensitivity analysis

All of the aforementioned analyses were based on the 35 cases who had complete data from before and after MDA. When the missing post-MDA data from the one egg-positive child were addressed using multiple imputation techniques, the changes in average scores and their distributions did not differ significantly, and interpretation of the results was unchanged (Supplemental Material, S1 File, Tables A-M).

## Discussion

The purpose of the present study was to pilot a standardized assessment tool, the BASC-2 Teacher Rating Scale, to assess the impact of praziquantel MDA on student behaviors in an area that is highly endemic for *S*. *mansoni*. In our sample of 35 Kenyan school-age children, all behaviors showed improvement after MDA, as assessed by composite scores both for children with high intensity infections and their schoolmates who were egg-negative (but likely carrying very low intensity *S*. *mansoni* infections [5, 8]). For the assessed composite domains of externalizing problems, internalizing problems, school problems, and the Behavioral Symptoms Index, there were statistically significant decreases in mean BASC-2 T scores following treatment intervention in one or both egg-status groups. Behaviors included in these categories are teacher-reported hyperactivity, aggression, conduct problems, anxiety, depression, somatization, attention problems, learning problems, withdrawal, and social stress [22]. The domain of adaptive skills also showed improvement after praziquantel, with statistically significant improvement in two of its subscales, social skills and study skills in the combined group of subjects. In general, children with *S*. *mansoni* egg-negative status had more favorable behavior scores, both before and after treatment, than those with egg-positive status; however, in multivariable analysis, only the differences in externalizing problems and overall BSI were found to be statistically significant between egg-status groups, independent of the timing of the assessment (Table 4).

The post-treatment change in the summary domain T scores was most substantial for school problems, which suggests that untreated schistosomiasis negatively impacts many students’ education. Our results also indicate that schistosomiasis may be associated with externalizing problems, as the mean domain scores were significantly greater among children with eggs compared to children without eggs pre-treatment, and were greater among egg-positive children before they were treated, as compared to their own post-treatment scores. The behaviors included in the externalizing problems domain are obvious act-out behaviors that are relatively easy for teachers to perceive, which may explain why differences in this domain may have been larger, such that they were statistically significant even with a limited sample size. Although the change in adaptive skills was marginal overall, the changes in social skills and study skills were significant following MDA. Such a change in study skills again suggests that schistosomiasis may have an important impact on educational success.

The observed post-treatment improvement in school problems and study skills is consistent with previous studies of the impact of schistosome infection on cognitive performance and school achievement, which have indicated that schistosomiasis is associated with a reduced ability to learn [14, 30]. Although school behavior is different from cognition, negative and positive behaviors could affect school performance in a way that is hard to distinguish from cognitive ability. In Tanzania, schoolchildren with schistosomiasis had significantly impaired short term memory and reaction times compared to uninfected children [31]. In Egypt, performance on intelligence tests was significantly lower in infected children [32]. In addition, in China, children’s cognitive function test scores, particularly for fluency, free recall, and picture search, was shown to be improved after praziquantel treatment [33]. Combined with the findings of the present study, these findings suggest that chronic schistosomiasis can continually impair children’s intellectual functioning and affect the ultimate success of their education. However, our study only examined treatment effects for children living in a *S*. *mansoni*-endemic area, and it is not certain that the same results would be obtained in a *S*. *haematobium*-endemic zone, or in an area with mixed infections.

A high level of heterogeneity in behavior score changes was observed among children in the initial egg-negative group, with post-treatment T scores not changing much for some, but changing dramatically for others. This likely reflects the fact that, in an *S*. *mansoni-*endemic zone, a child’s testing egg-negative does not necessarily exclude the presence of low-level chronic infection. Often, lower intensity infections go undetected in standard diagnostic tests [1, 5, 6, 8, 34]. Our post-treatment findings among egg-negative children make the important point that MDA may have a much wider impact on at-risk, school age children in schistosomiasis-endemic areas, i.e., MDA has a greater effect than that measured by only looking at treatment impacts for *S*. *mansoni* egg-positive children.

We have not investigated the biological basis of our results. Previous studies have demonstrated increases in schistosome antigen-specific production of immunoregulatory cytokines three weeks after treatment, although some cytokines, such as gamma interferon, remained unchanged [35]. Because cytokine levels can influence mood [19, 20], our results are consistent with a model in which reduced inflammation can improve emotional outlook, much in the way analgesics and antipyretics can improve the mood of a teething toddler. Additional studies will be needed to test this hypothesis and to evaluate further how intensity of schistosome infection has an impact on the magnitude of observed behavior problems.

There are limitations to our study. The sample size for this pilot study was small, with a total of 36 students, aged 8 to 11 years, from 6 different schools. Although both groups were well-matched with respect to age, sex and school distribution, with this study size we were unable to rule out the possibility that factors other than infection status could explain the observed outcomes. We feel that future studies, with increased sample sizes and age ranges, are now warranted to test our findings. In addition, we have only used the 129 question BASC-2 Teacher Rating Scale in our pilot study. Although each of the BASC-2 rating scales are independently validated, inclusion of the Parent Rating Scales (PRS) and Self-Report of Personality (SRP) in future studies would provide more comprehensive data to triangulate individual children’s behavioral status before and after anti-schistosomal therapy. The ability to adjust for covariates was limited in this study due to sample size, so that its findings are subject to potential bias due to confounding. In future studies, socioeconomic status (SES), including family income and parental education, should be included as a covariate, as SES is reported to significantly affect children’s behaviors [36]. Finally, the BASC-2 has been normalized to children in the United States and Latin America but not, specifically, to children in Kenya, where this study was performed. However, the instrument’s 13,000-person normalization sample included such a wide range of SES and races/ethnicities that we believe it was appropriate to use in our study location. Still, formal validation of the BASC-2 in Kenya should be a priority for future work.

To our knowledge, this was the first study to use quantitative survey tools to evaluate the behavior of children with schistosomiasis. Results of this study indicate a previously unacknowledged aspect of chronic *Schistosoma-*related morbidity, whose existence makes yet another argument in favor of MDA for control of schistosomiasis.

The results suggest that schistosomiasis negatively impacts the classroom behaviors of young Kenyan students, thus threatening their ability to maximize their education. This finding has significant implications for disease-endemic areas like western Kenya, where overt *S*. *mansoni* infection rates reach up to 70% [4]. If these findings are confirmed, this would provide an important rationale for the expansion of schistosomiasis control programs, particularly for regular MDA with praziquantel in locations with persistent transmission. In 2014, the World Health Organization reported only 34.6% coverage of school-age children needing treatment for schistosomiasis, falling far short of their recommended 75% target [2]. In addition to persuasive arguments for treating schistosomiasis to reduce childhood anemia, stunting, and undernutrition [6], this paper adds evidence that controlling schistosomiasis may be an essential component of achieving full childhood development and optimal educational attainment in *Schistosoma-*endemic countries.

## Supporting information

S1 File Results obtained with missing study data handled by multiple imputation. Tables A through M showing results of repeat statistical testing in which missing data were inferred by imputation.
(DOC)

S2 File Supporting data file. Anonymized individual demographic data and BASC-2 subscores for the 36 children enrolled in the study.
(XLSX)

S1 Table Mean BASC-2 T scores for *S*. *mansoni* egg-positive and egg-negative students before and after treatment with praziquantel. (DOC)

S2 Table Paired t-test for changes in individuals’ BASC-2 scores from before-treatment (X_1_) to after-treatment (X_2_), with egg-positive and egg-negative groups combined (N = 35). (DOC)

S3 Table Paired t-test for changes in individuals’ BASC-2 adaptive scale subscores from before-treatment (X_1_) to after-treatment (X_2_), with egg-positive and egg-negative groups combined (N = 35). (DOC)

S4 Table Paired t-test for changes in individuals’ BASC-2 scores following MDA in the group who were *S*. *mansoni* egg-positive before treatment (N = 17). (DOC)

S5 Table Paired t-test for changes in individuals’ BASC-2 scores following MDA in the group who were *S*. *mansoni* egg-negative before treatment (N = 18). (DOC)
