# Supplementary material for: Change in children’s school behavior after mass administration of praziquantel for Schistosoma mansoni infection in endemic areas of western Kenya: A pilot study using the Behavioral Assessment System for Children (BASC-2)
Source: PLoS One. 2017 Jul 26;12(7):e0181975. doi: 10.1371/journal.pone.0181975 (PMC5528892; doi:10.1371/journal.pone.0181975)
Supplement: S1 File — Tables A through M showing results of repeat statistical testing in which missing data were inferred by imputation. (DOC) [file pone.0181975.s001.doc]

**Results Obtained With Missing Study Data Handled By Multiple Imputation**

1. **Mean composite BASC-2 T Scores after treatment (Missing data inferred by i**mputation)

| **Infection Status** | **Mean (Std Dev)** | | | | |
| --- | --- | --- | --- | --- | --- |
|  | **Externalizing Problems** | **Internalizing Problems** | **School Problems** | **Behavioral Symptoms Index** | **Adaptive Skills** |
| **Egg Negative before MDA** | 52.22 (4.83) | 62.22 (10.69) | 49.94 (6.90) | 53.94 (6.92) | 49.61 (9.06) |
| **Egg Positive before MDA** | 56.44 (8.87) | 66.83 (13.67) | 51.75 (10.17) | 57.01 (10.01) | 47.10 (11.04) |

1. **Mean scores of BASC-2 subscales on Externalizing Problems (Missing data inferred by imputation)**

| **Composite Scale** | **Subscales** | **Mean (Std Dev)** | | | |
| --- | --- | --- | --- | --- | --- |
|  |  | **Before MDA** | | **After MDA** | |
|  |  | **Egg Positive** | **Egg Negative** | **Egg Positive**  **before MDA** | **Egg Negative before MDA** |
| **Externalizing Problems** |  | 61.72 (11.74) | 54.61 (8.17) | 56.44 (8.87) | 52.22 (4.83) |
|  | Hyperactivity | 59.17 (10.07) | 52.78 (6.20) | 54.43 (8.78) | 51.61 (4.77) |
|  | Aggression | 64.89 (13.53) | 56.44 (12.25) | 57.98 (8.43) | 54.56 (7.25) |
|  | Conduct Problems | 58.78 (12.98) | 53.50 (7.33) | 55.39 (13.41) | 50.06 (4.39) |

1. **Mean Scores of BASC-2 subscales on Internalizing Problems (Missing data inferred by imputation)**

| **Composite Scale** | **Subscales** | **Mean (Std Dev)** | | | |
| --- | --- | --- | --- | --- | --- |
|  |  | **Before MDA** | | **After MDA** | |
|  |  | **Egg Positive** | **Egg Negative** | **Egg Positive before MDA** | **Egg Negative before MDA** |
| **Internalizing Problem** |  | 70.83 (11.25) | 68.44 (13.32) | 66.83 (13.67) | 62.22 (10.69) |
|  | Anxiety | 62.17 (13.25) | 59.17 (13.00) | 60.70 (15.31) | 55.28 (9.00) |
|  | Depression | 60.56 (11.00) | 54.78 (9.32) | 58.52 (13.00) | 55.17 (7.16) |
|  | Somatization | 77.72 (10.87) | 80.44 (16.27) | 71.30 (16.17) | 68.94 (15.75) |
|  | Atypicality | 52.83 (9.67) | 52.78 (6.79) | 56.13 (13.64) | 53.50 (7.74) |
|  | Withdrawal | 55.61 (9.11) | 52.11 (11.88) | 56.28 (15.61) | 55.06 (12.12) |
|  |  |  |  |  |  |

1. **Mean Scores of BASC-2 subscales on School Problems (Missing data inferred by imputation)**

| **Composite Scale** | **Subscales** | **Mean (Std Dev)** | | | |
| --- | --- | --- | --- | --- | --- |
|  |  | **Before MDA** | | **After MDA** | |
|  |  | **Egg Positive** | **Egg Negative** | **Egg Positive before MDA** | **Egg Negative before MDA** |
| **School Problems** |  | 57.06 (12.26) | 54.72 (8.66) | 51.75 (10.17) | 49.94 (6.90) |
|  | Attention Problem | 52.83 (9.67) | 52.78 (6.79) | 49.75 (9.09) | 48.61 (6.71) |
|  | Learning Problem | 60.00(13.91) | 55.89 (9.69) | 53.60 (11.09) | 51.44 (7.02) |

1. **Mean Scores of BASC-2 subscales on Adaptive Skills (Missing data inferred by imputation)**

| **Composite Scale** | **Subscales** | **Mean (Std Dev)** | | | |
| --- | --- | --- | --- | --- | --- |
|  |  | **Before MDA** | | **After MDA** | |
|  |  | **Egg Positive** | **Egg Negative** | **Egg Positive before MDA** | **Egg Negative before MDA** |
| **Adaptive Skills** |  | 44.83 (8.90) | 46.83 (6.91) | 47.10 (11.04) | 49.61 (9.06) |
|  | Adaptability | 45.22 (6.44) | 45.39 (5.92) | 44.87 (9.43) | 46.00 (8.49) |
|  | Functional Communication | 41.50 (12.32) | 45.83 (9.69) | 42.81 (15.45) | 46.17 (9.90) |
|  | Social Skills | 47.28 (9.27) | 47.61 (7.96) | 51.65 (13.07) | 53.00 (10.66) |
|  | Leadership | 47.22 (8.58) | 48.33 (8.42) | 48.99 (11.51) | 51.00 (7.82) |
|  | Study Skills | 46.39 (10.89) | 48.67 (7.75) | 48.79 (15.45) | 52.00 (6.74) |

1. **Paired t-test for changes in individuals’ BASC-2 scores from before-treatment (
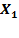
) to after-treatment (
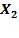
) (Missing data inferred by i**mputation)

| **Variable** | 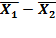 | **Std. Error of (**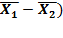 | **t** | **d.f.** | **p-value** |
| --- | --- | --- | --- | --- | --- |
| **Externalizing Problem** | 3.84 | 1.33 | 2.90 | 35 | **0.0064** |
| **Internalizing Problem** | 5.25 | 2.06 | 2.58 | 35 | **0.0142** |
| **School Problem** | 5.14 | 1.33 | 3.89 | 35 | **0.0004** |
| **BSI** | 2.77 | 1.36 | 2.06 | 35 | **0.0468** |
| **Adaptive Skill** | -2.47 | 1.34 | -1.87 | 35 | 0.0698 |

1. **P-values of Type 3 tests of the estimated fixed effects of time of testing, pre-treatment infection status, and time-status interaction (Missing data inferred by imputation)**

| **Effect** | **P Values** | | | | |
| --- | --- | --- | --- | --- | --- |
|  | Externalizing Problems | Internalizing Problems | School Problems | Behavioral Symptoms Index | Adaptive Skills |
| **Time** | **0.0176** | 0.2006 | **0.0132** | **0.0365** | 0.3338 |
| **Status** | **0.0027** | 0.5080 | 0.3317 | **0.0137** | 0.4088 |
| **Time*Status** | 0.3223 | 0.6030 | 0.8549 | 0.2984 | 0.8677 |

1. **Estimated fixed effects for Externalizing Problems (Missing data inferred by imputation)**

| **Effect** | **Status** | **Estimate** | **S.E.** | **DF** | **t** | **p-value** |
| --- | --- | --- | --- | --- | --- | --- |
| **Intercept** |  | 61.72 | 2.53 | 5 | 24.38 | <.0001 |
| **Time** | After | -5.28 | 2.20 | 34 | -2.40 | **0.0176** |
| **Status before MDA** | Egg Negative | -7.11 | 2.37 | 34 | -3.00 | **0.0027** |
| **Time*Status** | After*Egg Negative | 2.90 | 2.93 | 34 | 0.99 | 0.3223 |

1. **Estimated fixed effects for Internalizing Problems (Missing data inferred by imputation**)

| **Effect** | **Status** | **Estimate** | **S.E.** | **DF** | **t** | **p-value** |
| --- | --- | --- | --- | --- | --- | --- |
| **Intercept** |  | 70.83 | 3.28 | 5 | 21.62 | <.0001 |
| **Time** | After | -4.00 | 3.12 | 34 | -1.28 | 0.2006 |
| **Status before MDA** | Egg Negative | -2.39 | 3.61 | 34 | -0.66 | 0.5080 |
| **Time*Status** | After*Egg Negative | -2.22 | 4.27 | 34 | -0.52 | 0.6030 |

1. **Estimated fixed effects for School Problems (Missing data inferred by imputation)**

| **Effect** | **Status** | **Estimate** | **S.E.** | **DF** | **t** | **p-value** |
| --- | --- | --- | --- | --- | --- | --- |
| **Intercept** |  | 57.06 | 3.00 | 5 | 19.03 | <.0001 |
| **Time** | After | -5.30 | 2.13 | 34 | -2.49 | **0.0132** |
| **Status before MDA** | Egg Negative | -2.33 | 2.40 | 34 | -0.97 | 0.3317 |
| **Time*Status** | After*Egg Negative | 0.53 | 2.87 | 34 | 0.18 | 0.8549 |

1. **Estimated fixed effects for Behavioral Symptom Index (Missing data inferred by imputation)**

| **Effect** | **Status** | **Estimate** | **S.E.** | **DF** | **t** | **p-value** |
| --- | --- | --- | --- | --- | --- | --- |
| **Intercept** |  | 61.39 | 2.84 | 5 | 21.65 | <.0001 |
| **Time** | After | -4.38 | 2.08 | 34 | -2.10 | **0.0365** |
| **Status before MDA** | Egg Negative | -6.00 | 2.43 | 33 | -2.47 | **0.0137** |
| **Time*Status** | After*Egg Negative | 2.93 | 2.82 | 33 | 1.04 | 0.2984 |

1. **Estimated fixed effects for Adaptive Skills (Missing data inferred by imputation)**

| **Effect** | **Status** | **Estimate** | **S.E.** | **DF** | **t** | **p-value** |
| --- | --- | --- | --- | --- | --- | --- |
| **Intercept** |  | 44.83 | 2.67 | 5 | 16.82 | <.0001 |
| **Time** | After | 2.27 | 2.34 | 34 | 0.97 | 0.3338 |
| **Status before MDA** | Egg Negative | 2.00 | 2.42 | 34 | 0.83 | 0.4088 |
| **Time*Status** | After*Egg Negative | 0.51 | 3.06 | 34 | 0.17 | 0.8677 |

1. **Mean difference in the change in BASC-2 scores from before-treatment to after-treatment for both egg-positive and egg-negative groups (Missing data inferred by imputation)**

| **Variable** | **Mean Difference** | | **Standard Deviation** | | **95% Confidence Limits** |
| --- | --- | --- | --- | --- | --- |
|  | Egg Positive | Egg Negative | Egg Positive | Egg Negative |  |
| **Externalizing Problems** | 8.04 | 5.5 | 6.32 | 5.00 | (-4.36, 20.43) |
| **Internalizing Problems** | 10.62 | 11.78 | 7.54 | 6.70 | (-4.16, 25.40) |
| **School Problems** | 7.37 | 8.11 | 5.33 | 5.85 | (-3.08, 17.82) |
| **Behavioral Symptoms Index** | 7.62 | 6.56 | 5.28 | 4.44 | (-2.72, 17.97) |
| **Adaptive Skills** | 6.40 | 6.56 | 5.04 | 5.36 | (-3.48, 16.29) |
