# Supplementary material for: Change in children’s school behavior after mass administration of praziquantel for Schistosoma mansoni infection in endemic areas of western Kenya: A pilot study using the Behavioral Assessment System for Children (BASC-2)
Source: PLoS One. 2017 Jul 26;12(7):e0181975. doi: 10.1371/journal.pone.0181975 (PMC5528892; doi:10.1371/journal.pone.0181975)
Supplement: S1 Table — (DOC) [file pone.0181975.s003.doc]

**S1 Table. Mean BASC-2 T scores for students initially *S. mansoni* egg-positive or egg-negative on stool exam,** before and after treatment with praziquantel

| **Time** | **Infection Status** | **Mean (Std Dev)** | | | | |
| --- | --- | --- | --- | --- | --- | --- |
|  |  | **Externalizing Problems** | **Internalizing Problems** | **School Problems** | **Behavioral Symptoms Index** | **Adaptive Skills** |
| **Before treatment** | **Egg Negative** | 54.6 (8.2) | 68.4 (13.3) | 54.7 (8.7) | 55.4 (8.6) | 46.8 (6.9) |
|  | **Egg Positive** | 61.7 (11.7) | 70.8 (11.2) | 57.1 (12.3) | 61.4 (11.4) | 44.8 (8.9) |
| **After treatment** | **Egg Negative before MDA** | 52.2 (4.8) | 62.2 (10.7) | 49.9 (6.9) | 53.9 (6.9) | 49.6 (9.1) |
|  | **Egg Positive before MDA** | 56.7 (7.9) | 66.6 (13.3) | 51.7 (9.8) | 57.5 (9.7) | 46.7 (10.0) |
