# Supplementary material for: Change in children’s school behavior after mass administration of praziquantel for Schistosoma mansoni infection in endemic areas of western Kenya: A pilot study using the Behavioral Assessment System for Children (BASC-2)
Source: PLoS One. 2017 Jul 26;12(7):e0181975. doi: 10.1371/journal.pone.0181975 (PMC5528892; doi:10.1371/journal.pone.0181975)
Supplement: S2 Table — (DOC) [file pone.0181975.s004.doc]

**S2 Table. Paired t-test for changes in individuals’ BASC-2 scores from before-treatment (X_1_) to after-treatment (X_2_), with egg-positive and egg-negative groups combined (N=35)**

| **Variable** | **Mean**  **difference**  **(X_1_ – X_2_)** | **Standard deviation of differences** | **P-Value** | **Cohen Effect Size** |
| --- | --- | --- | --- | --- |
| **Externalizing Problems** | 4.00 | 7.96 | **0.0054** | 0.5025 (Medium) |
| **Internalizing Problems** | 5.37 | 12.26 | **0.0139** | 0.4380 (Small) |
| **School Problems** | 5.26 | 7.96 | **0.0004** | 0.6608 (Medium) |
| **BSI** | 2.89 | 8.10 | **0.0425** | 0.3570 (Small) |
| **Adaptive Skills** | -2.43 | 7.92 | 0.0784 | 0.3068 (Small) |
