# Supplementary material for: Change in children’s school behavior after mass administration of praziquantel for Schistosoma mansoni infection in endemic areas of western Kenya: A pilot study using the Behavioral Assessment System for Children (BASC-2)
Source: PLoS One. 2017 Jul 26;12(7):e0181975. doi: 10.1371/journal.pone.0181975 (PMC5528892; doi:10.1371/journal.pone.0181975)
Supplement: S3 Table — (DOC) [file pone.0181975.s005.doc]

**S3 Table. Paired t-test for changes in individuals’ BASC-2 Adaptive Scale subscores from before-treatment (X_1_) to after-treatment (X_2_), with egg-positive and egg-negative groups combined (N=35)**

| **Variable** | **Mean**  **difference**  **(X_2_ – X_1_)** | **Standard deviation of differences** | **P-Value** | **Effect Size** |
| --- | --- | --- | --- | --- |
| **Adaptability** | -0.09 | 7.46 | 0.9462 | 0.0121 |
| **Social Skills** | 4.29 | 10.39 | **0.0200** | 0.4130 (Small) |
| **Leadership** | 2.69 | 9.22 | 0.0940 | 0.2918 (Small) |
| **Study Skills** | 2.74 | 7.77 | **0.0444** | 0.3526 (Small) |
| **Functional Communication** | 0.77 | 10.58 | 0.6689 | 0.0728 |
