# Supplementary material for: Change in children’s school behavior after mass administration of praziquantel for Schistosoma mansoni infection in endemic areas of western Kenya: A pilot study using the Behavioral Assessment System for Children (BASC-2)
Source: PLoS One. 2017 Jul 26;12(7):e0181975. doi: 10.1371/journal.pone.0181975 (PMC5528892; doi:10.1371/journal.pone.0181975)
Supplement: S4 Table — (DOC) [file pone.0181975.s006.doc]

**S4 Table. Paired t-test for changes in individuals’ BASC-2 scores following MDA in the group who were *S. mansoni* egg-positive before treatment (N=17)**

| **Variable** | **Mean**  **difference**  **(X_1_ – X_2_)** | **Standard deviation of differences** | **P-Value** | **Effect Size** |
| --- | --- | --- | --- | --- |
| **Externalizing Problems** | 5.71 | 8.62 | **0.0149** | 0.6624  (Medium) |
| **Internalizing Problems** | 4.47 | 12.55 | 0.1611 | 0.3562 (Small) |
| **School Problems** | 5.76 | 7.03 | **0.0038** | 0.8193 (Large) |
| **Behavioral Symptoms Index** | 4.41 | 8.23 | **0.0420** | 0.5358 (Medium) |
| **Adaptive Skills** | -2.06 | 7.92 | 0.2995 | 0.2601 (Small) |
