# Supplementary material for: Change in children’s school behavior after mass administration of praziquantel for Schistosoma mansoni infection in endemic areas of western Kenya: A pilot study using the Behavioral Assessment System for Children (BASC-2)
Source: PLoS One. 2017 Jul 26;12(7):e0181975. doi: 10.1371/journal.pone.0181975 (PMC5528892; doi:10.1371/journal.pone.0181975)
Supplement: S5 Table — (DOC) [file pone.0181975.s007.doc]

**S5 Table. Paired t-test for changes in individuals’ BASC-2 scores following MDA in the group who were *S. mansoni* egg-negative before treatment (N=18)**

| **Variable** | **Mean**  **difference**  **(X_1_ – X_2_)** | **Standard deviation of differences** | **P-Value** | **Effect Size** |
| --- | --- | --- | --- | --- |
| **Externalizing Problems** | 2.39 | 7.14 | 0.1737 | 0.3347 (Small) |
| **Internalizing Problems** | 6.22 | 12.28 | **0.0462** | 0.5065 (Medium) |
| **School Problems** | 4.78 | 8.93 | **0.0365** | 0.5352 (Medium) |
| **Behavioral Symptoms Index** | 1.44 | 7.94 | 0.4505 | 0.1814 |
| **Adaptive Skills** | -2.78 | 8.13 | 0.1653 | 0.3419 (Small) |
